# Polyethylene glycol-polyester based temperature-sensitive hydrogel delivering mesenchymal stem cell-derived exosomes enhances acute skin wound healing

**DOI:** 10.3389/fbioe.2025.1730631

**Published:** 2025-11-19

**Authors:** Zining Wei, Jie Ren, Jianshe Hu, Haiming Wei

**Affiliations:** 1 State Key Laboratory of Immune Response and Immunotherapy, Division of Life Sciences and Medicine, University of Science and Technology of China, Hefei, China; 2 Center for Molecular Science and Engineering, College of Science, Northeastern University, Shenyang, China

**Keywords:** wound healing, PLGA-PEG-PLGA, thermosensitive hydrogel, mesenchymal stem cells, exosomes

## Abstract

Skin wound healing remains a significant clinical challenge. Conventional dressings have limitations in maintaining an optimal wound microenvironment and preventing secondary injury. In this study, we developed a Poly (lactic-co-glycolic acid)-poly (ethylene glycol)-poly (lactic-co-glycolic acid) (PLGA-PEG-PLGA, PPP) thermosensitive hydrogel loaded with mesenchymal stem cell-derived exosomes (MSC-Exos) to enhance acute skin wound healing by prolonging exosome retention and bioavailability at the wound site. The hydrogel exhibited a rapid sol-gel transition at approximately 32 °C, demonstrating good mechanical stability (storage modulus (G′) > loss modulus (G″)) and self-healing properties at physiological temperature. *In vitro* experiments revealed that PPP/Exos showed superior biocompatibility with L929 mouse fibroblast cells (L929 cells) and human umbilical vein endothelial cells (HUVECs), significantly promoting cell proliferation and vascular tube formation. In a Sprague-Dawley (SD) rat full-thickness skin defect model, the PPP/Exos group markedly accelerated wound closure. By day 14, wound closure reached 98.6% in the PPP/Exos group, compared with 87.6% in the control group. Histopathological examination further revealed that PPP/Exos treatment effectively enhanced granulation tissue formation, attenuated inflammatory responses, facilitated re-epithelialization, and substantially increased collagen deposition. Through immunohistochemical analysis, we identified three mechanisms underlying the enhanced wound healing: promoted angiogenesis, accelerated myofibroblast differentiation, and reduced inflammation. Collectively, the PPP/Exos thermosensitive hydrogel, with its excellent biocompatibility, injectability, and sustained exosome release characteristics, significantly promotes wound healing through synergistic “angiogenesis-tissue remodeling-anti-inflammation” effects. This system offers a promising therapeutic strategy for clinical wound management and establishes a solid foundation for applications in regenerative medicine.

## 1 Introduction

As the largest organ of the human body, the skin serves as the primary barrier against physical, chemical, and biological insults from the external environment. When its structural integrity is compromised by injury, a sophisticated physiological repair cascade is immediately initiated to restore tissue architecture and function (Xu et al., 2015; Chen et al., 2024b). However, conventional wound dressings such as cotton bandages and gauze show limited efficacy in maintaining an appropriate wound microenvironment during clinical practice. Moreover, these traditional materials pose considerable risks of mechanical stripping and secondary trauma to nascent tissue during dressing changes (Xu et al., 2020; Chen et al., 2024a). Hydrogel dressings, by contrast, possess highly cross-linked three-dimensional network structures that confer exceptional water retention capacity, thereby creating a consistently moist environment that facilitates tissue regeneration (Xue et al., 2019; Wang L. et al., 2022; Chen et al., 2025). These materials also exhibit favorable oxygen permeability, excellent tissue compatibility, and substantial drug-loading capacity, making them well-suited for addressing the multifaceted requirements of complex wound management (Cheng et al., 2021; Wang Y. et al., 2022; Zhao et al., 2022). Thermosensitive hydrogels are particularly noteworthy, as they remain fluid at ambient temperature but undergo rapid transition to a semi-solid gel state under physiological conditions. This unique behavior enables precise conformity to wounds with irregular geometry while minimizing mechanical stress on regenerating tissues (Wu et al., 2024; Yang et al., 2024).

Among various polymeric systems for fabricating thermosensitive hydrogels, biodegradable block copolymers based on PEG combined with polyesters or polypeptides have attracted considerable attention due to their well-defined sol-gel phase transition near physiological temperature (approximately 37 °C) (Li et al., 2016; Patel et al., 2016). Polyester-PEG-polyester triblock amphiphilic copolymers have emerged as particularly promising candidates in this field, owing to their straightforward one-step synthesis, tunable phase transition behavior, and excellent biodegradability (Shi et al., 2021; Dethe et al., 2022; Guo et al., 2024). The gelation mechanism of these materials is primarily driven by their amphiphilic molecular architecture: at lower temperatures, the copolymers self-assemble into nanoscale micelles, which undergo further physical cross-linking as temperature rises above the lower critical solution temperature (LCST), ultimately establishing a continuous hydrogel network through reversible sol-gel transformation (Gong et al., 2009; Wang et al., 2017; Ge et al., 2023).

Recent advances highlight that the therapeutic efficacy of MSC-Exos is largely mediated by specific miRNAs and proteins (Lee et al., 2021). For instance, miR-21 can downregulate PTEN and activate PI3K/AKT signaling to enhance keratinocyte migration and proliferation, while miR-146a targets IRAK1/TRAF6 to dampen NF-κB activity and attenuate pro-inflammatory cytokine production, thereby shortening the inflammatory phase (Qin et al., 2023; Yang et al., 2023). In parallel, exosomal proteins such as vascular endothelial growth factor (VEGF) and Transforming growth factor beta (TGF-β) contribute to angiogenesis and matrix remodeling, coordinating granulation tissue formation and tissue maturation (Bian et al., 2022; Geng et al., 2022; Song et al., 2023). Compared with direct cell transplantation, exosomes offer distinct advantages regarding immunogenic risk, formulation stability, and supply chain feasibility. They can be preserved long-term through cryopreservation at −80 °C or lyophilization, enabling convenient storage and transportation (Varderidou-Minasian and Lorenowicz, 2020). Despite their broad application prospects in regenerative medicine, exosomes suffer from limited bioavailability due to rapid *in vivo* clearance and short half-life. This limitation becomes particularly pronounced during the protracted wound healing process, substantially compromising their therapeutic efficacy (Zhao et al., 2023). Therefore, developing multifunctional wound dressings capable of sustained and controlled exosome release represents a highly promising solution. For example, Lu et al. enhanced vascularized bone regeneration in a diabetic periodontal bone defect model by loading MSCs-Exos into a temperature-sensitive hydrogel composed of PEG-PPG-PEG copolymer. (Lu et al., 2025). In addition, Song et al. developed a temperature-sensitive hydrogel loaded with M2 macrophage-derived exosomes (M2^Exo^-loaded HP hydrogel) to alleviate the symptoms of osteoarthritis (OA) (Song et al., 2024).

Given the restricted local bioavailability of MSC-Exos resulting from brief *in vivo* exposure and rapid clearance, we developed a PEG-based thermosensitive triblock copolymer hydrogel with PLGA as the backbone (PLGA-PEG-PLGA) for loading and delivering MSC-Exos, thereby constructing a PLGA-PEG-PLGA/MSC-Exos (PPP/Exos) composite hydrogel. Our objective was to prolong exosome retention at wound sites and enhance their wound healing efficacy. This study conducted comprehensive characterization of PPP/Exos, including its physicochemical properties and sol-gel transition behavior near physiological temperature. *In vitro*, we employed HUVECs migration and tube formation assays to assess its pro-angiogenic potential. *In vivo*, we established a full-thickness skin defect model in SD rats to validate the wound healing-promoting effects of PPP/Exos and elucidate the underlying mechanisms.

## 2 Materials and methods

### 2.1 Materials

Glycolide (GA, ≥99% purity) and DL-lactide (LA, ≥98% purity) were procured from Innochem. Polyethylene glycol (PEG, MW = 1500, ≥99% purity) and tin (II) 2-ethylhexanoate [Sn(Oct)_2_, ≥99.8% purity] were obtained from Sigma Aldrich. Toluene was supplied by Sinopharm Chemical Reagent Co., Ltd. MSC-Exos were purchased from Huizhi Cell Industry Technology Innovation Research Co., Ltd. Deionized water was generated in-house using a laboratory water purification system. Prior to use, toluene underwent azeotropic reflux with sodium wire to remove residual moisture. All other solvents employed in this work were used as received without additional purification.

### 2.2 Methods

#### 2.2.1 Preparation of PPP/Exos

The PPP triblock copolymer was synthesized using a ring-opening polymerization method, with a monomer-to-initiator (M:I) ratio of 20:1, an L-lactide (LA)/glycolide (GA) ratio of 7:1, and PEG with a molecular weight of 1500 as the macro-initiator. Prior to polymerization, PEG1500 was subjected to strict pretreatment: it was wrapped in aluminum foil to protect it from light, purged with nitrogen, dried under vacuum at 150 °C and 4.5 mmHg for 3 h, and then cooled to room temperature. LA (0.07 mol, 10.08 g), GA (0.01 mol, 1.16 g), pretreated PEG1500 (0.004 mol, 6.00 g), and a toluene solution of tin (II) 2-ethylhexanoate [Sn(Oct)_2_] (0.1 mol L^-1^, 168 μL) as catalyst were added into a dried polymerization tube. The system was then evacuated and purged with nitrogen three times to completely remove toluene and air from the polymerization tube. Subsequently, the tube was sealed under high vacuum (<5 mmHg) and melt polymerization was carried out at 150 °C with magnetic stirring for 12 h. Upon completion of the reaction, the crude product was washed three times with deionized water at 80 °C to remove residual monomers and oligomers. The purified polymer was freeze-dried, placed in a sealed container, and stored at −20 °C protected from light until further use.

The PPP triblock copolymer was dissolved in phosphate-buffered saline (PBS, pH 7.4) at 4 °C to achieve a 25% (w/v) concentration, yielding the thermosensitive PPP hydrogel. To fabricate the PPP/Exos composite hydrogel, lyophilized MSC-Exos powder was incorporated into the PPP hydrogel at a final concentration of 200 μg/mL.

#### 2.2.2 Characterization

The chemical structure of the copolymer was elucidated by proton nuclear magnetic resonance spectroscopy (^1^H NMR, Bruker ARX 600, Germany) and Fourier-transform infrared spectroscopy (FT-IR, PerkinElmer Spectrum One (B), United States). Differential scanning calorimetry (DSC, Netzsch DSC-204, Germany) was employed to determine the thermodynamic properties. Scanning electron microscopy (SEM, HITACHI SU8010, Japan) allowed visualization of the material’s microstructural morphology.

### 2.3 Sol-gel transition of PPP hydrogel

The sol-gel transition temperature of the hydrogel was determined using the tube inversion method. Briefly, vials containing 0.5 mL of sample solution were pre-cooled in a water bath at 4 °C for 30 min. The temperature was then raised incrementally at 1 °C intervals. After each temperature increment, the vials were allowed to equilibrate and subsequently inverted. The gelation endpoint was defined as the temperature at which no flow was observed within 30 s upon inversion. All measurements were performed in triplicate and averaged.

### 2.4 Rheological properties of PPP/Exos hydrogels

Rheological properties were evaluated using an MCR302 rheometer (Anton Paar) equipped with 25 mm diameter parallel plate geometry and a gap distance of 0.5 mm. Temperature sweep experiments were conducted from 20 °C to 45 °C at a heating rate of 0.5 °C/min. Frequency sweep measurements were performed at 37 °Cwith a fixed shear strain of 0.5% over an angular frequency range of 1–100 rad/s (logarithmic scale). Strain sweep tests were carried out at 37 °C and a constant angular frequency of 10 rad/s, with shear strain varying from 0.1% to 1000% (logarithmic scale) to identify the linear viscoelastic region. The self-recovery behavior was assessed through cyclic step-strain tests at 37 °C and 10 rad/s, alternating the strain between 1% and 300% for five consecutive cycles.

### 2.5 *In vitro* biocompatibility of PPP/Exos hydrogels

Human umbilical vein endothelial cells (HUVECs) and L929 cells obtained from our laboratory were maintained in Dulbecco’s Modified Eagle Medium (DMEM, high glucose, Sigma, United States) supplemented with 10% fetal bovine serum (FBS, VivaCell, Shanghai, China) and 1% penicillin-streptomycin (Gibco, USA). Cultures were incubated at 37 °C in a humidified atmosphere containing 5% CO_2_.

Hydrogel extracts were prepared by immersing 10 mg of each hydrogel formulation in 1 mL of complete culture medium at 37 °C for 24 h, after which the supernatants were collected. L929 and HUVECs (5 × 10^4^ cells·mL^-1^) were seeded into 96-well plates at 100 μL per well. Following cell attachment, the medium was replaced with Control, PPP, or PPP/Exos extracts, and cells were cultured for an additional 24 h. CCK-8 working solution (110 μL per well, diluted 1:10) was then added and incubated for 2 h. Absorbance was measured at 450 nm to assess cell proliferation.

### 2.6 HUVECs scratch and tube formation assays

Six-well plates were pre-marked by drawing at least five parallel horizontal lines per well (spaced approximately 0.5–1 cm apart) with a marker and ruler on the underside of the plates. HUVECs in logarithmic growth phase were harvested by PBS washing, trypsinization, and centrifugation at 250 *g* for 5 min, followed by resuspension in medium. Cells (2 × 10^5^ in 2 mL medium) were seeded per well. Three experimental groups were prepared: Control, Gel, and Exo/Gel, each with three replicates, and incubated at 37 °C in a 5% CO_2_ atmosphere. Upon reaching confluence, a scratch was created perpendicular to the pre-marked lines in each well using a 200 μL pipette tip and ruler. Detached cells were removed by gentle washing with PBS, after which the corresponding conditioned media were added according to experimental grouping. Cells were further incubated for 12 h. Images of wound areas at the marked line positions were captured at 0 and 12 h under ×40 magnification.

To evaluate the pro-angiogenic potential of the hydrogels, we performed a Matrigel-based tube formation assay. Matrigel matrix (250 μL per well) was dispensed into 24-well plates and allowed to solidify at 37 °C for 60 min. HUVECs in logarithmic growth phase were harvested by routine trypsinization, centrifuged at 250 *g* for 5 min, and resuspended to a final density of 2 × 10^5^ cells·mL^-1^. Cell suspension (500 μL per well) was then seeded onto the pre-gelled Matrigel surface. After cell adhesion, cultures were treated with Control, PPP, or PPP/Exos extracts for 6 h. After calcein staining, tubular network formation was captured using an inverted fluorescence microscope at ×100 magnification from randomly selected fields. Quantitative analysis of junction numbers and total tube length was conducted using ImageJ software to assess angiogenic capacity.

### 2.7 *In vivo* experiments

All animal experiments were carried out according to the ARRIVE guidelines in accordance with National Research Council’s Guide for the Care and Use of Laboratory Animals (eighth edition), and were performed under the approval of the Experimental Animal Management Committee of University of Science and Technology of China (USTCACUC25120125110). All SD rats used in the experiment (male, 230–250 g, 6–8 weeks old) were purchased from Beijing Huafukang Biotechnology Co., Ltd. Upon arrival at the animal facility, the rats were housed under controlled environmental conditions at 20 °C–5 °C and 50%–60% relative humidity, with a strict 12 h light/12 h dark cycle, for an acclimation period of 7 days. The rats were housed individually, provided with standard laboratory-grade pellet feed and fresh drinking water, and kept in clean, well-ventilated cages. Body weight and general condition were monitored daily, and individual records were kept to ensure the animals recovered and adapted in a quiet and stable environment, thereby preparing them adequately for subsequent experiments.

Following a 3-day acclimatization period, animals were randomly allocated into three groups: Control, PPP, and PPP/Exos (n = 6 per group). Randomization was performed using SPSS software based on body weight ranking after ear tagging and initial weighing upon arrival.

A full-thickness dorsal skin defect model was created in Sprague-Dawley (SD) rats using bilateral symmetric wounds (10 mm diameter). Animals were anesthetized via intraperitoneal injection of 1% sodium pentobarbital (50 mg/kg) prior to wound creation. In the PPP, and PPP/Exos groups, the corresponding thermosensitive hydrogel was injected directly into the wound and covered with 3M Tegaderm dressing, which was changed every 3 days. Wound photographs were taken and recorded on days 0, 3, 7, 10, and 14. Wound areas were measured and analyzed using ImageJ software. The wound healing rate was calculated according to the following formula:

$$\text{Wound healing}=\frac{\left(S_{0}-S_{n}\right)}{S_{0}}\times 100\%$$

where S_0_ and S_n_ represent the initial wound area and the wound area at different time points, respectively.

### 2.8 Histology and immunohistochemistry

At 7 and 14 days postoperatively, SD rats from each group were anesthetized via intraperitoneal injection of 1% sodium pentobarbital (50 mg/kg). After confirming loss of consciousness and absence of pain response by pressing the toes with fingers or forceps without eliciting a reaction, the animals were euthanized by exsanguination through the abdominal aorta. Full-thickness wound tissues were collected and fixed in 10% neutral buffered formalin for 24 h, followed by dehydration through graded ethanol series and paraffin embedding. Serial sections of 5 μm thickness were subsequently prepared. Hematoxylin and eosin (H&E) staining was performed to examine histomorphological changes. Masson’s trichrome staining assessed collagen deposition, with ImageJ software employed to quantify the percentage of blue-stained area as an indicator of collagen remodeling. To evaluate neovascularization, immunohistochemistry (IHC) was conducted on tissue sections from corresponding time points. CD31 antibody was used to label vascular endothelial cells, while α-SMA antibody identified vascular smooth muscle cells. Whole-slide images were acquired using an Aperio CS2 digital scanner. Three randomly selected fields per section were analyzed to determine CD31-positive vessel density and the percentage of stained area.

### 2.9 Statistical analysis

Data are presented as mean ± standard deviation (SD). Statistical significance was evaluated using one-way analysis of variance (ANOVA). Differences were considered statistically significant at *p < 0.05, **p < 0.01, and ***p < 0.001.

## 3 Results and discussion

### 3.1 Preparation and characterization of PPP

**FIGURE 1:**
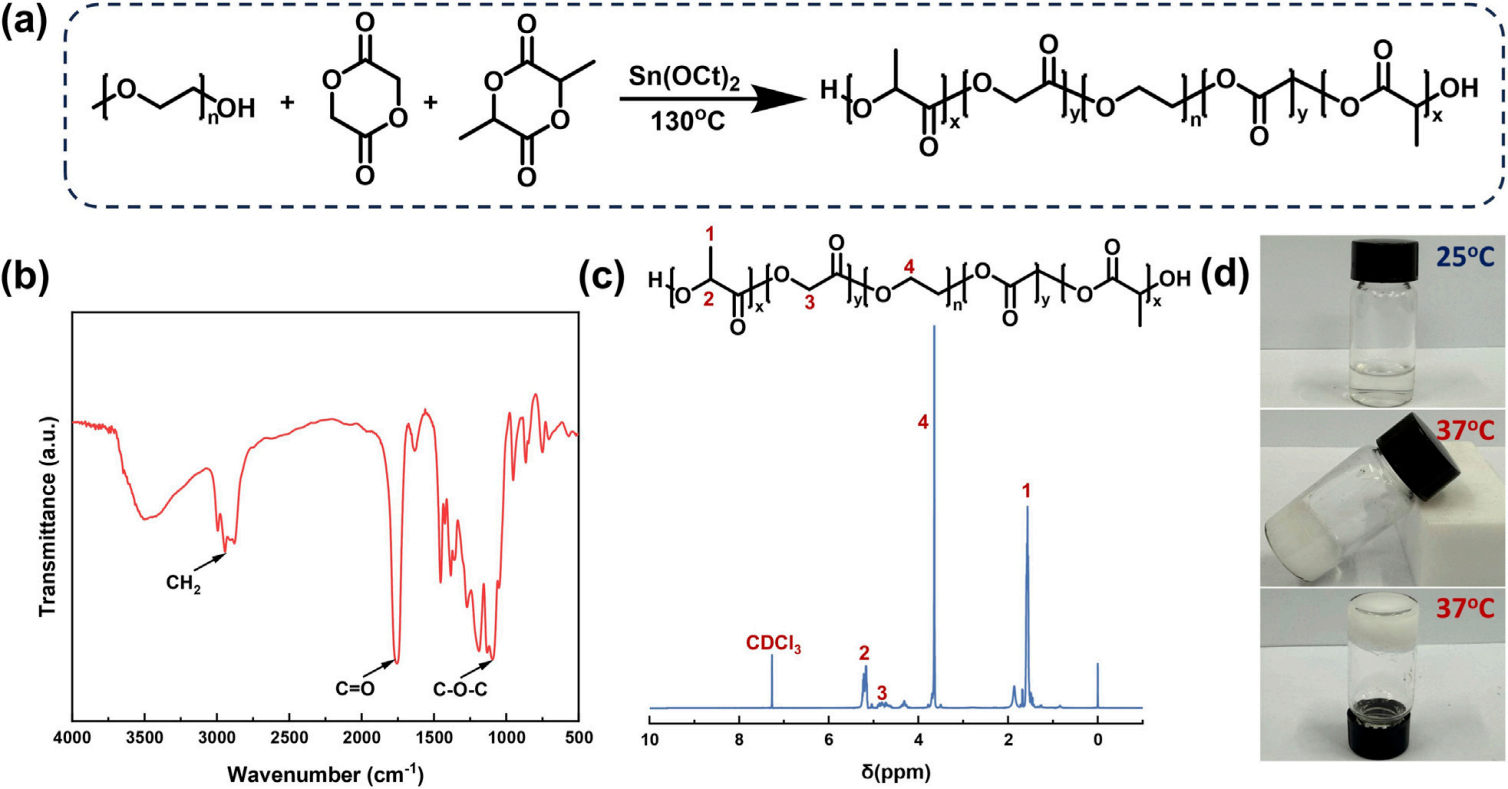
**(a)** Synthetic scheme of PPP. **(b)** FT-IR spectrum and **(c)** ^1^H NMR spectrum of PPP. **(d)** Photographs of PPP hydrogel at 25 °C and 37 °C.

The synthesis route of PPP is illustrated in Figure 1a. The triblock copolymer was prepared via bulk ring-opening polymerization of GA and LA using PEG1500 as the macroinitiator and Sn(Oct)_2_ as the catalyst. The chemical structure of PPP was confirmed by FT-IR and ^1^H NMR spectroscopy. As shown in Figure 1b, the FT-IR spectrum displayed characteristic absorption bands: the peak at 1758 cm^-1^ was attributed to the stretching vibration of C=O bonds in the polymer backbone, the band at 1096 cm^-1^ corresponded to C-O-C stretching of the PEG segment, and the peak at 2944 cm^-1^ was assigned to C-H stretching vibrations of CH_2_ groups. These spectral features collectively confirmed successful synthesis of the PPP copolymer. ^1^H NMR spectroscopy further elucidated the molecular structure of the synthesized copolymer (Figure 1c). Chemical shifts were assigned as follows: δ 1.57 ppm (-OCH(CH_3_)CO-), δ 3.64 ppm (-OCH_2_CH_2_-), δ 4.31 ppm (-OCH_2_CH_2_OCOCH_2_O-), δ 4.81 ppm (-OCH_2_CO-), and δ 5.17 ppm (-OCH(CH_3_)CO-). These assignments were consistent with the designed molecular structure of the PPP copolymer (Yu et al., 2008). PPP hydrogel was prepared at a concentration of 25% (w/v), and its sol-gel transition temperature was determined using the tube inversion method (Figure 1d) to evaluate gelation behavior. Results demonstrated that the PPP hydrogel underwent sol-gel transition at 37 °C.

### 3.2 Gel transition and rheological properties of hydrogels

**FIGURE 2:**
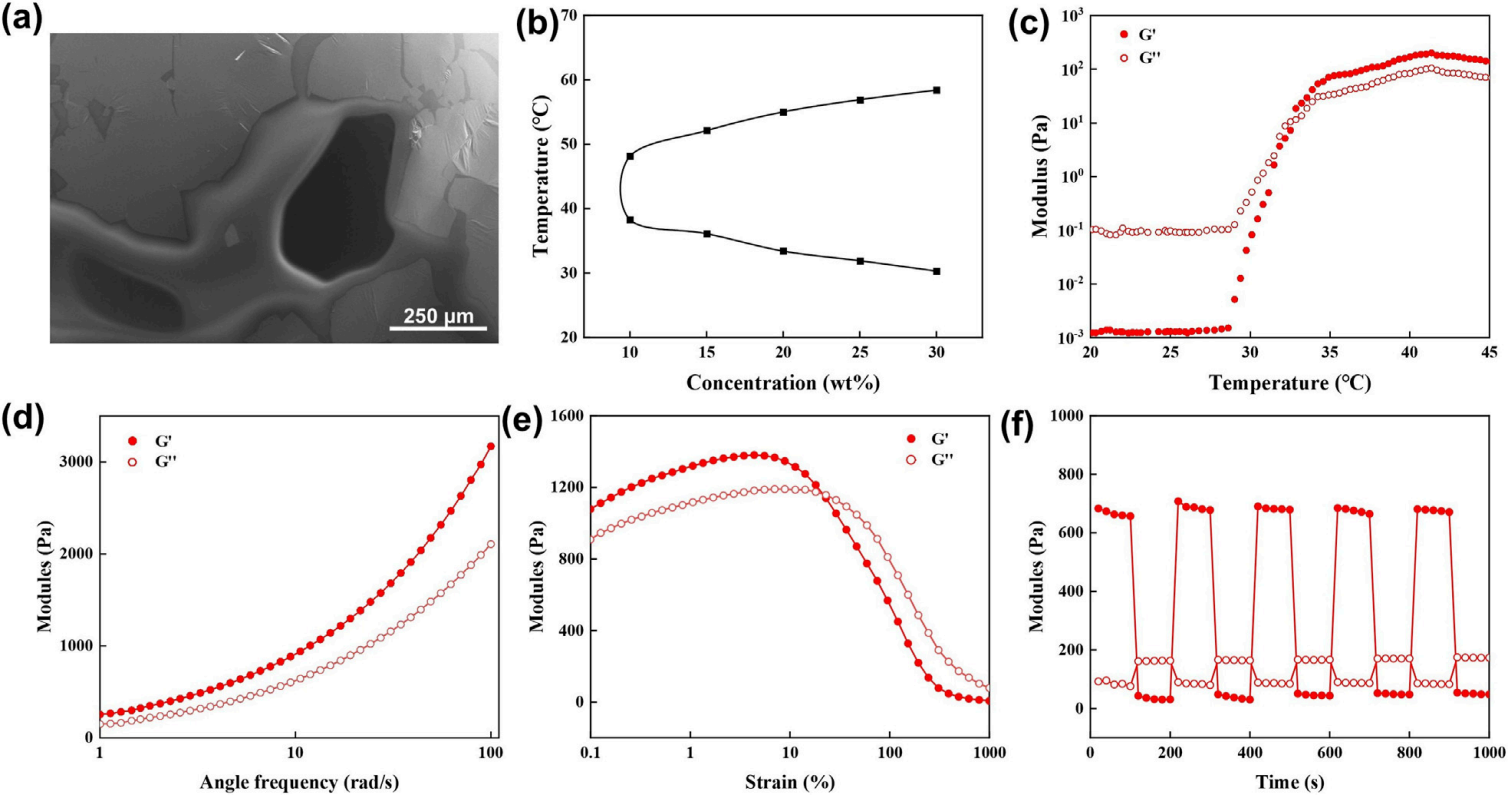
**(a)** SEM image of PPP hydrogel. **(b)** Phase diagram of PPP hydrogels at various concentrations as a function of temperature determined by the tube inversion method. **(c)** Temperature-dependent storage modulus (G′) and loss modulus (G″) of PPP/Exos hydrogel. **(d)** Frequency sweep, **(e)** strain sweep, and **(f)** consecutive step-strain measurements of PPP/Exos hydrogel at 37 °C.

PPP hydrogel exhibited a classical porous morphology characteristic of hydrogel networks, facilitating nutrient exchange between the hydrogel and wound tissue (Figure 2a). As shown in Figure 2b, the PPP hydrogel underwent a temperature-dependent phase transition, exhibiting three distinct physical states: sol-gel-precipitation. The sol-gel transition temperature of the PPP hydrogel decreased gradually with increasing PPP concentration. By adjusting the polymer ratio in the hydrogel, the sol-gel transition temperature could be precisely controlled within the range of 31 °C–32 °C, which is below physiological body temperature. Systematic characterization was performed to investigate the microstructure and rheological properties of the thermosensitive hydrogel. SEM imaging (Figure 2a) revealed that PPP/Exos hydrogel possessed a typical three-dimensional interconnected porous network with relatively uniform pore size distribution, which favors mass diffusion and transport. The temperature-concentration phase diagram (Figure 2b) demonstrated distinct lower critical solution temperature (LCST) behavior. Within the concentration range of 10–30 wt%, phase transition temperatures occurred between 30 °C–60 °C and reduced with polymer concentration, providing a theoretical foundation for tuning thermosensitive properties. Temperature sweep rheological measurements (Figure 2c) showed that during heating, G′ and G″ intersected at approximately 32 °C. Beyond this point, G′ increased sharply and exceeded G″, indicating sol-gel transition and formation of a stable three-dimensional network structure. Frequency sweep analysis (Figure 2d) revealed that G′ consistently exceeded G″ across the frequency range of 1–100 rad/s, with both moduli increasing modestly with frequency. This behavior confirmed typical elastic solid characteristics and excellent mechanical stability at 37 °C. Strain sweep experiments (Figure 2e) demonstrated that G′ and G″ remained essentially constant within the strain range of 0.1%–10%, defining the linear viscoelastic region. When strain exceeded approximately 10%, G′ began to decline, signifying network disruption and shear-thinning behavior. Cyclic step-strain testing (Figure 2f) revealed rapid and reversible transitions in G′ between low (1%) and high (300%) strain amplitudes. After five consecutive cycles, the hydrogel retained over 90% modulus recovery, demonstrating superior self-healing capacity.

### 3.3 *In vitro* cell proliferation and endothelial tubulogenesis assay of PPP/Exos hydrogel

**FIGURE 3:**
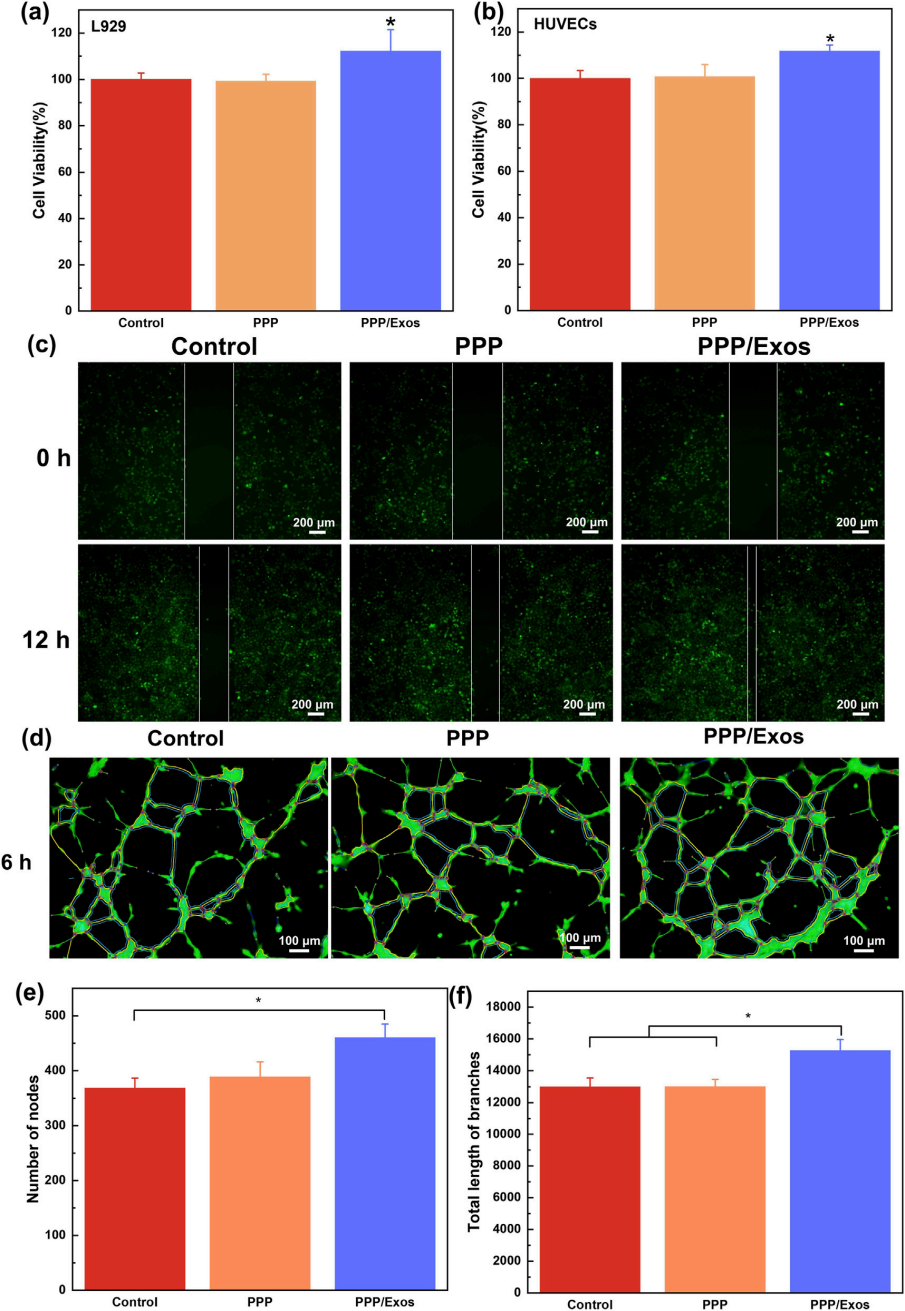
Biocompatibility of hydrogels evaluated in **(a)** L929 cells and **(b)** HUVECs. **(c)** Images of HUVECs migration and tube formation assays **(d)** Representative images of tube formation assay. Quantification of **(e)** number of junctions and **(f)** total branch length. Each value is presented as mean ± SD (n = 3). *p < 0.05, **p < 0.01, ***p < 0.001.

To assess the biocompatibility and pro-angiogenic potential of the thermosensitive hydrogel, cytotoxicity assays and tube formation experiments were conducted using L929 cells and HUVECs, respectively. CCK-8 assay results (Figures 3a,b) revealed that cell viability remained above 95% for both L929 and HUVECs cultured with PPP hydrogel, showing no significant difference compared to the control group. This confirms excellent biocompatibility without cytotoxic effects. Notably, the PPP/Exos group exhibited significantly enhanced cell viability reaching approximately 110%, indicating that encapsulated MSC-Exos effectively promoted cell proliferation. This enhancement can be attributed to the abundance of bioactive molecules within exosomes, including growth factors and microRNAs, which positively regulate cellular metabolism and proliferation. In the HUVEC migration assay, the average scratch healing distance (△d) of each group was measured after 12 h (Figure 3c). The △d of the control group was 40.75 ± 0.97 μm, and that of the PPP group was comparable (40.44 ± 5.90 μm), with no statistically significant difference between the two groups (P > 0.05). In contrast, the cell migration ability of the PPP/Exos group was significantly enhanced, with a △d of 70.78 ± 8.70 μm, which was significantly higher than that of the control group (P < 0.01). These results indicate that the PPP/Exos hydrogel can effectively promote HUVEC migration. To further evaluate the impact on neovascularization, an *in vitro* tube formation assay was performed using HUVECs seeded on Matrigel. Fluorescence microscopy images showed that all three groups formed characteristic tubular network structures after 6 h incubation (Figure 3d). However, quantitative analysis demonstrated that the PPP/Exos group displayed significantly higher junction numbers and total vessel length compared to both control and PPP groups (Figures 3e,f). These findings suggest that while PPP hydrogel alone exerts minimal influence on vessel formation, the PPP/Exos composite markedly promotes endothelial cell migration, junction formation, and tubulogenesis, demonstrating superior pro-angiogenic activity. This angiogenic effect may be related to pro-angiogenic factors carried by exosomes, such as VEGF and basic fibroblast growth factor (bFGF), along with miRNAs that regulate angiogenesis-related gene expression. Collectively, these results demonstrate that the PPP/Exos composite hydrogel possesses not only favorable biocompatibility but also significantly enhances cell proliferation and neovascularization, establishing a solid foundation for its application in tissue engineering, wound repair, and treatment of ischemic diseases.

### 3.4 Evaluation of the efficacy of PPP/Exos hydrogel in wound healing

**FIGURE 4:**
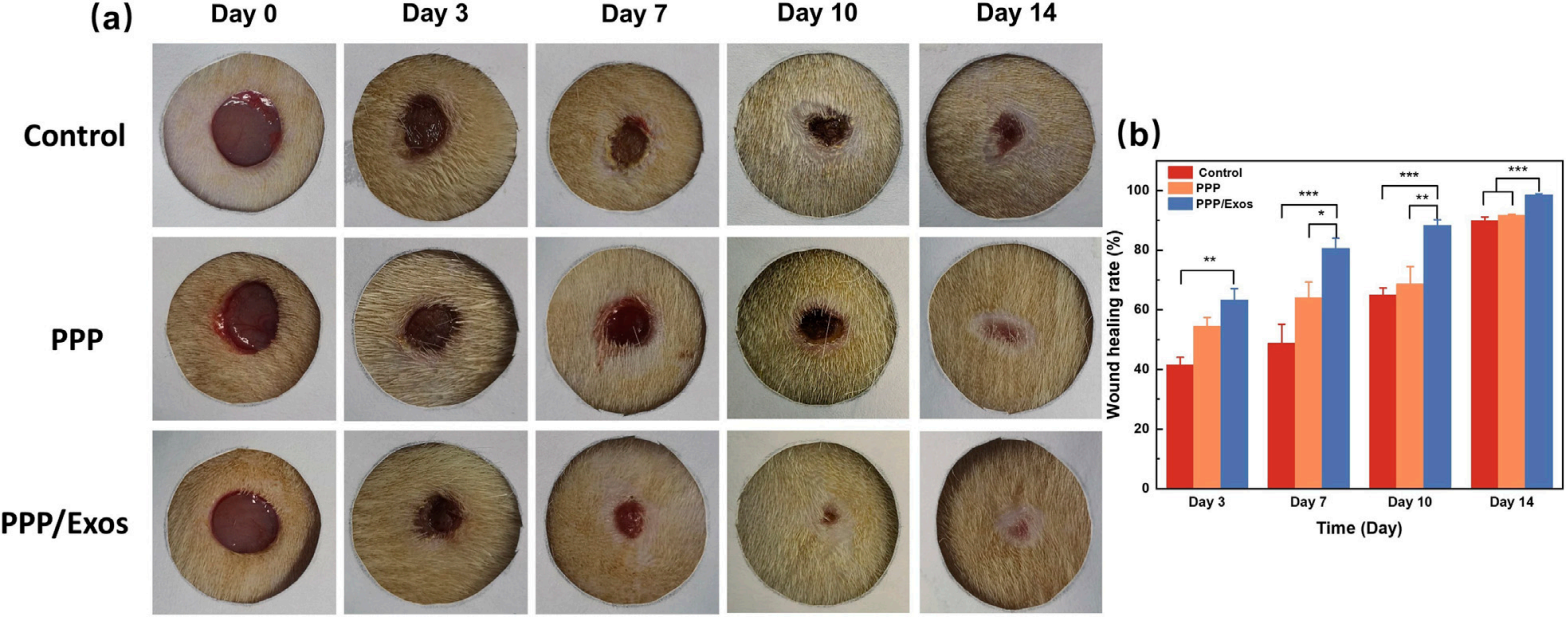
**(a)** Representative macroscopic images of wound healing. **(b)** Quantification of wound closure rates at different time points. Each value is presented as mean ± SD (n = 3). *p < 0.05, **p < 0.01, ***p < 0.001.

To evaluate the *in vivo* wound healing capacity of the thermosensitive hydrogel, a full-thickness skin defect model was established in Sprague-Dawley rats, and the repair efficacy of different materials was systematically assessed. As shown in Figure 4a, all three groups exhibited progressive wound contraction and epithelialization during the 14-day observation period, though healing rates differed markedly. The Control group maintained large open wounds with visible dark red granulation tissue on day 3, and although substantial reduction occurred by day 14, complete closure was not achieved. The PPP group showed no apparent difference from controls during the early phase (day 3), but demonstrated moderate healing promotion starting from day 7, with newly formed epithelium migrating from the periphery toward the center. Notably, the PPP/Exos group displayed optimal wound healing throughout the entire observation period, exhibiting accelerated wound contraction. By day 7, wound area was substantially reduced, and by day 14, near-complete closure was achieved with pale regenerated epithelial tissue visible at the wound center. Quantitative analysis (Figure 4b) further validated these observations. On day 3, the PPP/Exos group achieved a wound closure rate of approximately 63.2%, significantly exceeding the 41.4% observed in controls, indicating early therapeutic effects of MSC-Exos. By day 7, intergroup differences became more pronounced: the PPP/Exos group reached 80.6% closure, significantly surpassing both the PPP group (64.0%) and Control group (48.7%), demonstrating that PPP/Exos hydrogel markedly enhanced healing capacity. Data from days 10 and 14 showed that the PPP/Exos group maintained the highest healing rates at 88.3% and 98.6%, respectively, both significantly superior to the PPP group (68.7% and 91.8%) and Control group (approximately 64.9% and 87.6%). These results indicate that PPP/Exos thermosensitive hydrogel exerts sustained pro-healing effects throughout all wound healing phases. The superior healing performance of the PPP/Exos group can be attributed to several mechanisms: (1) the *in situ* gelation property of PPP hydrogel provides a moist healing environment that minimizes wound dehydration and infection risk; (2) the abundance of growth factors (such as VEGF, TGF-β, and EGF) and regulatory microRNAs within exosomes promotes fibroblast proliferation, collagen synthesis, angiogenesis, and epithelial cell migration (Tutuianu et al., 2021); (3) the anti-inflammatory and immunomodulatory functions of exosomes help shorten the inflammatory phase and accelerate the repair process (Arabpour et al., 2021).

### 3.5 Histological evaluation of wound regeneration

**FIGURE 5:**
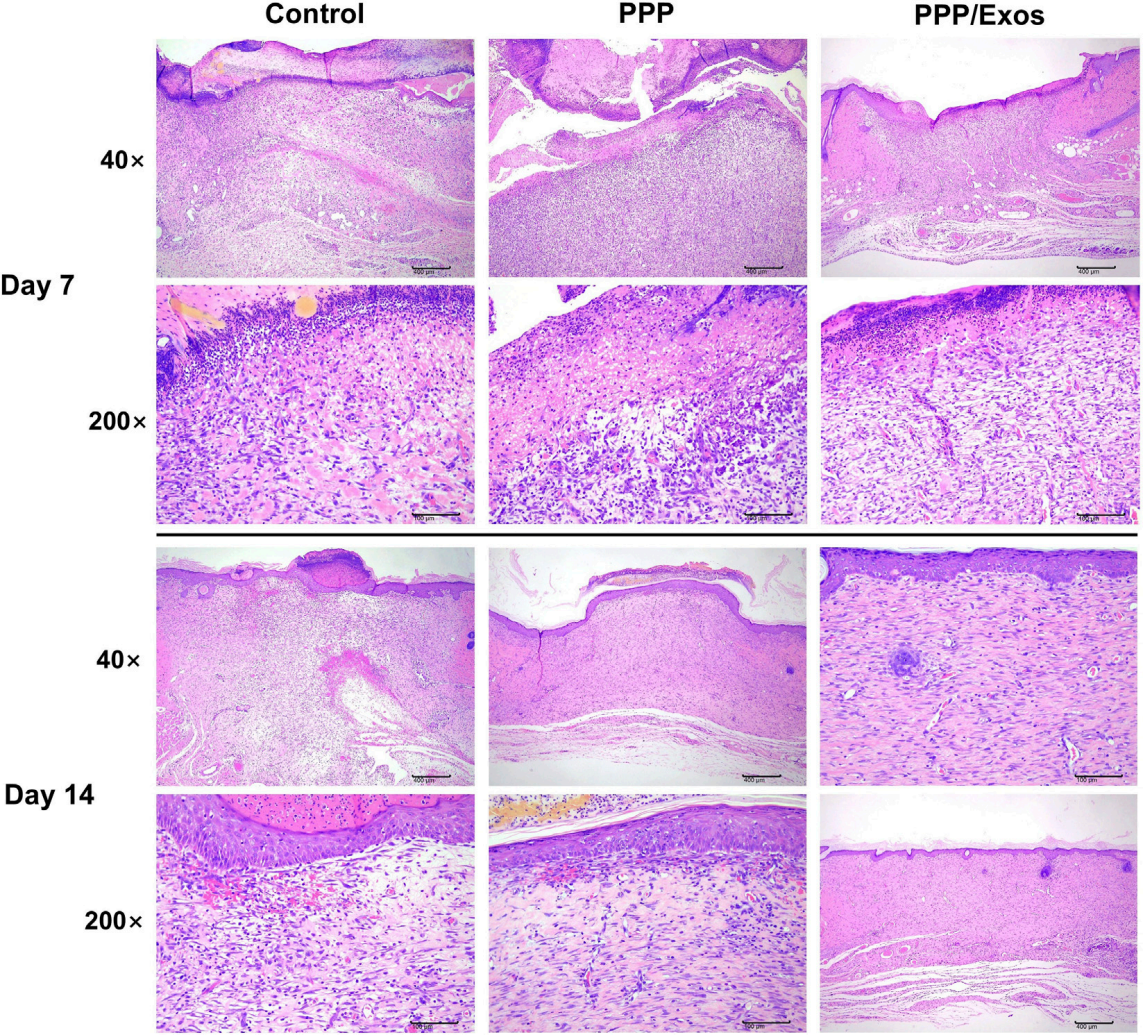
H&E staining of wound tissue sections from different treatment groups at days 7 and 14.

To evaluate the wound repair quality from a histopathological perspective, skin tissues harvested on day 7 were subjected to H&E staining and light microscopy examination (Figure 5). Pathological analysis revealed that all groups were in the inflammatory-proliferative transition phase, exhibiting multiple characteristic pathological features. Regarding ulceration, focal ulcers of mild to moderate severity were observed in 50% (3/6) of Control samples, 33.3% (2/6) of PPP samples, and 33.3% (2/6) of PPP/Exos samples. These ulcers were characterized by epidermal loss, dermal necrosis with cellular debris formation, tissue edema, and inflammatory cell infiltration predominantly consisting of neutrophils with lobulated nuclei and mononuclear cells, indicating wounds remained in an active inflammatory phase. Dermal inflammatory responses were present across all groups, with mild to moderate multifocal or diffuse inflammatory cell infiltration observed in 50% (3/6), 66.7% (4/6), and 66.7% (4/6) of samples in the Control, PPP, and PPP/Exos groups, respectively, primarily comprising mononuclear cells and neutrophils. Notably, all samples (6/6, 100%) displayed mild to moderate granulation tissue proliferation, characterized by markedly increased numbers of fibroblasts and fibrocytes with oval and elongated spindle-shaped nuclei, abundant weakly eosinophilic cytoplasm, and prominent capillary neovascularization—an important indicator of wound progression into the proliferative repair phase. Hemorrhage was observed in 66.7% (4/6), 66.7% (4/6), and 33.3% (2/6) of samples in the Control, PPP, and PPP/Exos groups, respectively, manifesting as mild to minimal red blood cell extravasation. The significantly lower hemorrhage incidence in the PPP/Exos group suggests superior hemostatic and tissue repair capacity. Eschar formation occurred at rates of 50% (3/6), 33.3% (2/6), and 16.7% (1/6) in the Control, PPP, and PPP/Exos groups, respectively. The eschar, composed of necrotic cells, neutrophils, and edema fluid, was least prevalent in the PPP/Exos group, indicating better wound bed cleanliness and superior healing quality. Overall, day 7 pathological features revealed that the PPP and PPP/Exos groups exhibited reduced ulcer areas compared to controls, improved inflammatory control, and more complete granulation tissue filling, demonstrating that the thermosensitive hydrogel and exosome loading significantly promoted early wound repair. By day 14, wound repair progression had accelerated substantially across all groups, with histological architecture approaching normalization, though intergroup differences persisted. Regarding ulcer repair, ulcerated regions in the Control, PPP, and PPP/Exos groups were largely replaced by newly formed fibroblasts and fibrocytes, with increasingly organized cellular arrangement in the dermis, enhanced collagen fiber deposition, and maturing tissue architecture. Inflammatory responses diminished markedly, with significantly reduced inflammatory cell infiltration and a cellular shift from predominantly neutrophils in early stages to mainly mononuclear cells, indicating resolution of acute inflammation and transition to tissue remodeling. For epidermal repair, the epidermal layer in all groups essentially restored continuity and exhibited reactive hyperplasia, manifested as mild epidermal thickening, increased spinous cell layers (pronounced epidermal thickening visible in 1/6 Control samples), and progressive stratum corneum formation, indicating near-complete re-epithelialization. However, some Control and PPP samples retained residual eschar or focal hemorrhage, suggesting wound repair had not yet reached optimal status, whereas the PPP/Exos group demonstrated more complete tissue remodeling and epidermal repair, with smooth wound surfaces, absence of visible eschar residue, and tissue architecture more closely resembling normal skin—healing quality markedly superior to Control and PPP groups. Comprehensive day 14 pathological evaluation indicates that PPP/Exos thermosensitive hydrogel effectively promotes granulation tissue maturation, accelerates epidermal re-epithelialization, facilitates inflammatory resolution, and enhances wound healing quality, significantly reducing the time required for complete wound closure.

**FIGURE 6:**
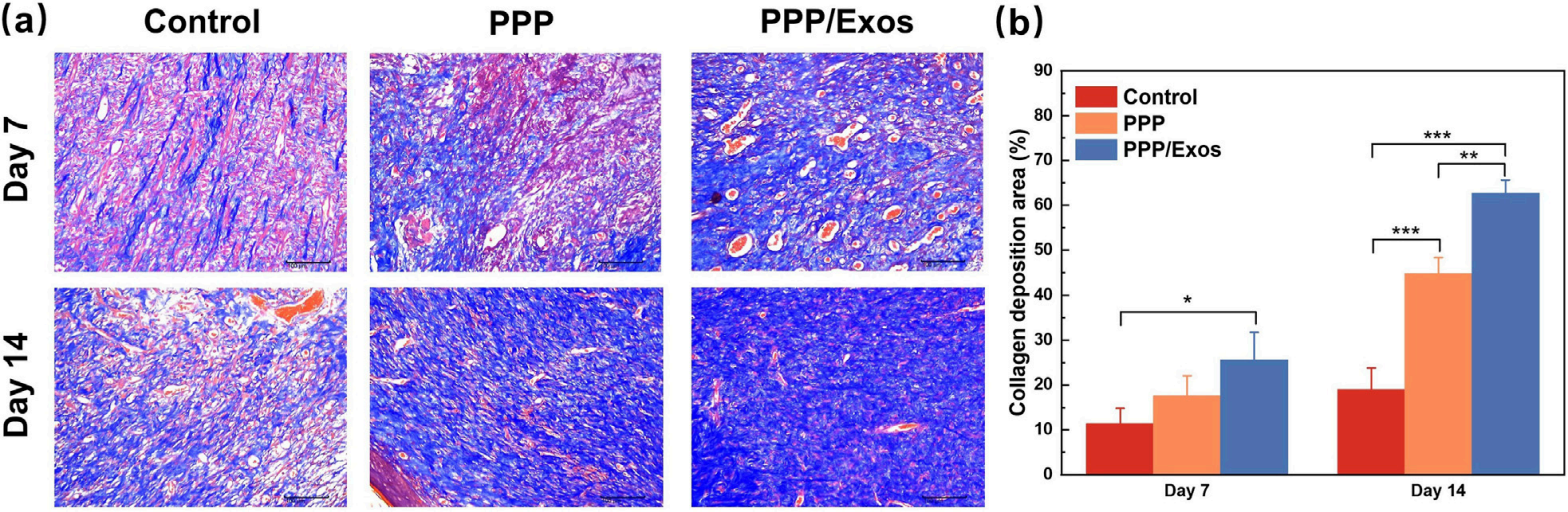
**(a)** Representative Masson’s trichrome staining images of wound tissue sections from different treatment groups at days 7 and 14. **(b)** Quantitative analysis of collagen deposition. Each value is presented as mean ± SD (n = 3). *p < 0.05, **p < 0.01, ***p < 0.001.

To assess collagen deposition and extracellular matrix (ECM) remodeling during wound healing, Masson’s trichrome staining was performed on skin tissue sections harvested at days 7 and 14 (Figure 6). As shown in representative images, collagen fibers were stained blue, while nuclei and cytoplasm appeared red. At day 7, all three groups exhibited relatively sparse and disorganized collagen fiber distribution, indicating the early phase of ECM reconstruction. Notably, the PPP/Exos group displayed more prominent blue-stained areas compared to controls, suggesting accelerated collagen synthesis. Quantitative analysis revealed that the collagen deposition area in the PPP/Exos group (25.5% ± 6.2%) was significantly higher than in the Control group (11.4% ± 3.43%), while the PPP group (17.6% ± 3.5%) showed an increasing trend without reaching statistical significance. By day 14, collagen fiber deposition increased substantially in all groups, presenting more compact, orderly, and parallel-arranged fiber architectures, particularly evident in the PPP and PPP/Exos groups. Quantitative results demonstrated that both the PPP group (44.8% ± 3.5%) and PPP/Exos group (62.7% ± 3.0%) had significantly greater collagen deposition areas than the Control group (19.0% ± 4.8%). Furthermore, the PPP/Exos group exhibited significantly higher collagen deposition than the PPP group, indicating that MSC-Exos markedly promoted collagen synthesis and ECM maturation. These findings demonstrate that PPP/Exos hydrogel not only provides a three-dimensional scaffold conducive to cell infiltration and proliferation but also stimulates fibroblast activation, enhances collagen production, and promotes tissue remodeling, thereby improving the quality and mechanical strength of regenerated skin tissue.

**FIGURE 7:**
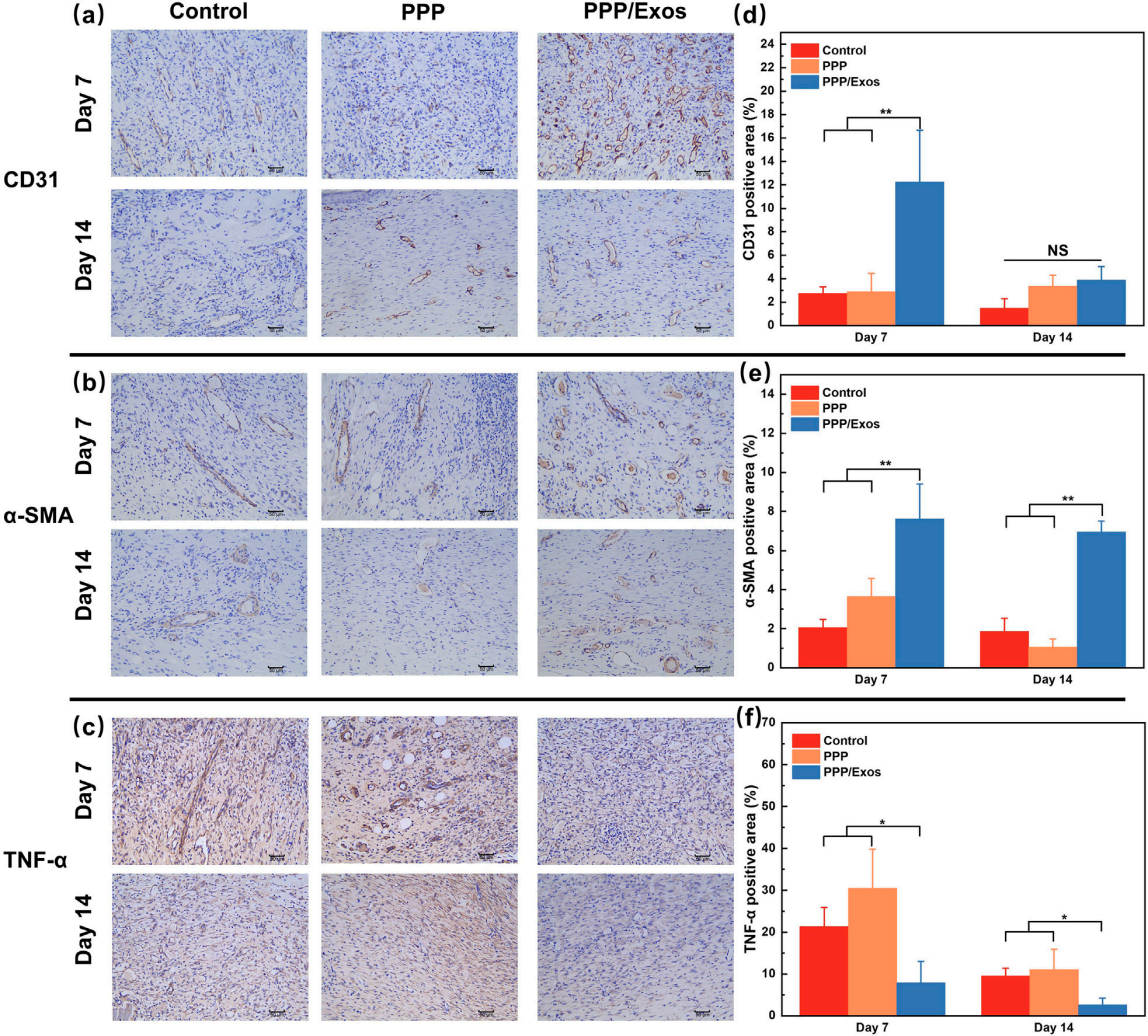
**(a–c)** Representative immunohistochemical (IHC) staining images. **(d)** Quantification of CD31-positive areas. **(e)** Quantification of α-SMA-positive areas. **(f)** Quantification of TNF-α-positive areas. Each value is presented as mean ± SD (n = 3). *p < 0.05, **p < 0.01, ***p < 0.001.

To further elucidate the molecular mechanisms underlying PPP/Exos hydrogel-mediated wound healing promotion, immunohistochemical staining was employed to systematically assess the expression of angiogenic marker CD31 and myofibroblast marker α-SMA (Mayrand et al., 2012). CD31 expression analysis revealed that angiogenesis plays a pivotal role in wound repair. As shown in Figure 7a, at day 7, CD31-positive staining in the Control and PPP groups displayed scattered distribution with weak signals presenting as pale brown coloration, indicating limited neovascularization. In contrast, the PPP/Exos group exhibited abundant dark brown positive staining areas, with vascular endothelial cells arranged in continuous tubular structures showing denser and more uniform tissue distribution. Quantitative analysis (Figure 7d) demonstrated that CD31-positive expression area in the PPP/Exos group at day 7 (12.2% ± 4.4%) significantly exceeded both the Control (2.7% ± 0.6%) and PPP groups (2.9% ± 1.5%), indicating that exosomes markedly promoted early-stage angiogenesis. By day 14, CD31 expression levels declined across all groups—a pattern consistent with the physiological processes of vascular maturation and remodeling during late-stage wound healing. Quantitative results showed that the PPP/Exos group maintained significantly higher CD31-positive area (3.9% ± 1.1%) compared to Controls (1.5% ± 0.8%), while the PPP group (3.4% ± 0.9%) showed elevation without reaching statistical significance. The narrowed intergroup differences at day 14 suggest that vascular networks had matured and stabilized. These findings demonstrate that PPP/Exos hydrogel significantly promotes angiogenesis during the critical early phase of wound healing (day 7), providing adequate oxygen and nutrient supply for tissue repair, followed by facilitation of vascular network maturation and functional refinement during the remodeling phase. Analysis of α-SMA (myofibroblast marker) expression further revealed the tissue remodeling-promoting effects of PPP/Exos. As illustrated in Figure 7b, at day 7, Control group samples displayed weak and sparsely distributed α-SMA-positive staining, with slight increases in the PPP group, whereas the PPP/Exos group exhibited pronounced dark brown positive staining predominantly distributed along wound margins and within granulation tissue. Positive cells displayed spindle-shaped morphology with orderly arrangement, indicating substantial fibroblast differentiation into contractile myofibroblasts. Quantitative analysis (Figure 7e) demonstrated that α-SMA-positive expression area in the PPP/Exos group at day 7 (7.6% ± 1.8%) significantly surpassed the Control group (2.0% ± 0.4%), while the PPP group (3.7% ± 0.9%) showed elevation without statistical significance. By day 14, α-SMA expression increased across all groups, consistent with the physiological processes of wound contraction and collagen remodeling. Quantitative results revealed that the PPP/Exos group (6.9% ± 0.9%) maintained significantly higher α-SMA expression than both Control (2.1% ± 0.6%) and PPP groups (1.4% ± 0.6%), with significant differences between PPP and PPP/Exos groups, indicating that exosomes continuously promoted myofibroblast differentiation and functional maintenance. Myofibroblasts serve as principal effector cells for wound contraction and ECM remodeling; their elevated expression facilitates accelerated wound closure, enhanced tissue mechanical strength, and promoted directional alignment of collagen fibers. As shown in Figures 7c,f on Day 7, the control group exhibited the strongest TNF-α–positive signals within the wound area, while the PPP/Exos group showed significantly lower levels (p < 0.05). This indicates that the loading of MSC-Exos can effectively suppress the proinflammatory response during the early phase of wound healing. By Day 14, TNF-α expression was further reduced in all three groups, but the PPP/Exos group remained significantly lower than the control group (p < 0.05), suggesting that its anti-inflammatory effect is sustained. In line with known mechanisms, MSC-Exos may promote the transition of macrophages from the M1 to M2 phenotype by delivering miR-146a/miR-21 and inhibiting NF-κB signaling, thereby accelerating the progression from inflammation to the proliferative phase. Consistent with the enhanced early angiogenesis and subsequent improvement in tissue remodeling observed in this study, these results indicate that PPP/Exos possess advantages in immune regulation.

Collectively, these results demonstrate that PPP/Exos hydrogel orchestrates an integrated “triple synergy” mechanism involving robust early-stage angiogenesis, sustained myofibroblast-mediated tissue remodeling, and effective suppression of inflammatory responses. The hydrogel’s ability to provide a sustained release platform for exosomes enables precise temporal regulation: it accelerates vascular network formation and modulates inflammation during the early healing phase while continuously promoting collagen remodeling and wound contraction throughout later stages. This coordinated cascade not only expedites wound closure but also leads to improved tissue organization and superior repair quality, underscoring the material’s potential for enhancing complex wound healing through dynamic and multifaceted molecular regulation.

## 4 Conclusion

This study successfully constructed a multifunctional wound dressing based on MSC-Exos-loaded PLGA-PEG-PLGA thermosensitive hydrogel (PPP/Exos), which exhibits ideal thermosensitive phase transition properties, good mechanical stability, and self-healing capacity, enabling minimally invasive injection and precise conformity to irregularly shaped wounds. *In vitro* investigations confirmed that PPP/Exos hydrogel possesses excellent biocompatibility and significantly promotes fibroblast and vascular endothelial cell proliferation as well as tubular network formation. *In vivo* studies using a rat full-thickness skin defect model demonstrated that the PPP/Exos group exhibited markedly superior wound closure rates compared to controls. Histopathological analysis revealed that this system achieves high-quality wound repair through effective modulation of inflammatory responses, promotion of granulation tissue formation, acceleration of re-epithelialization, and substantial enhancement of collagen deposition. This platform holds promise for addressing critical limitations of conventional wound treatment strategies and demonstrates excellent clinical translation potential.

## Data Availability

The raw data supporting the conclusions of this article will be made available by the authors, without undue reservation.
